# Best practice guidelines for citizen science in mental health research: systematic review and evidence synthesis

**DOI:** 10.3389/fpsyt.2023.1175311

**Published:** 2023-09-08

**Authors:** Olamide Todowede, Felix Lewandowski, Yasuhiro Kotera, Alison Ashmore, Stefan Rennick-Egglestone, Doreen Boyd, Stuart Moran, Kristin Berre Ørjasæter, Julie Repper, Dan Robotham, Michael Rowe, Dafni Katsampa, Mike Slade

**Affiliations:** ^1^School of Health Sciences, Institute of Mental Health, University of Nottingham, Nottingham, United Kingdom; ^2^School of Psychology, University of Nottingham, Nottingham, United Kingdom; ^3^University of Nottingham Libraries, Nottingham, United Kingdom; ^4^School of Geography, University of Nottingham, Nottingham, United Kingdom; ^5^Information Services, University of Nottingham, Nottingham, United Kingdom; ^6^Nord University, Faculty of Nursing and Health Sciences, Health and Community Participation Division, Namsos, Norway; ^7^ImROC, Nottinghamshire Healthcare NHS Foundation Trust, Nottingham, United Kingdom; ^8^McPin Foundation, London, United Kingdom; ^9^Program for Recovery and Community Health, Yale University, New Haven, CT, United States; ^10^National Elf Service, London, United Kingdom; ^11^School of Psychology, University of Hertfordshire, Hatfield, United Kingdom

**Keywords:** mental health, citizen science, community science, best practice guideline, public engagement

## Abstract

Partnering with people most affected by mental health problems can transform mental health outcomes. Citizen science as a research approach enables partnering with the public at a substantial scale, but there is scarce guidance on its use in mental health research. To develop best practise guidelines for conducting and reporting research, we conducted a systematic review of studies reporting mental health citizen science research. Documents were identified from electronic databases (*n* = 10), grey literature, conference proceedings, hand searching of specific journals and citation tracking. Document content was organised in NVIVO using the ten European Citizen Science Association (ECSA) citizen science principles. Best practise guidelines were developed by (a) identifying approaches specific to mental health research or where citizen science and mental health practises differ, (b) identifying relevant published reporting guidelines and methodologies already used in mental health research, and (c) identifying specific elements to include in reporting studies. A total of 14,063 documents were screened. Nine studies were included, from Australia, Belgium, Canada, Denmark, Netherlands, Spain, the UK, and the United States. Citizen scientists with lived experience of mental health problems were involved in data collection, analysis, project design, leadership, and dissemination of results. Most studies reported against some ECSA principles but reporting against these principles was often unclear and unstated. Best practise guidelines were developed, which identified mental health-specific issues relevant to citizen science, and reporting recommendations. These included citizen science as a mechanism for empowering people affected by mental health problems, attending to safeguarding issues such as health-related advice being shared between contributors, the use of existing health research reporting guidelines, evaluating the benefits for contributors and impact on researchers, explicit reporting of participation at each research stage, naming the citizen science platform and data repository, and clear reporting of consent processes, data ownership, and data sharing arrangements. We conclude that citizen science is feasible in mental health and can be complementary to other participatory approaches. It can contribute to active involvement, engagement, and knowledge production with the public. The proposed guidelines will support the quality of citizen science reporting.

## Introduction

Globally, mental ill health accounts for at least 18% of the global disease burden, with an estimated global annual cost projected at US $6 trillion by 2030 (1, 2). Mental health remains a neglected priority, with underinvestment by policymakers and funders (3). A secondary analysis of longitudinal cohort study data has demonstrated that levels of mental health distress in the population increased further during the COVID-19 pandemic (4).

Mental health systems have been criticised for insufficient attention to human rights, (5) an over-emphasis on medical approaches, (6) and for ignoring structural inequalities (7). There are calls for disruptive innovations to transform mental health and social care systems, (8) for example by harnessing the experiential knowledge of people who live or have lived with mental health problems (9–12). Involving people with lived experience of mental health conditions in service delivery, development and leadership can promote equity, citizenship, and social justice, challenge the status quo, and strengthen systems (12). Public involvement in mental health research has benefits for those who participate; it can support personal empowerment, recovery and social connectivity, and can promote positive mental health outcomes (13).

Citizen science is an emerging approach to enabling public involvement at scale, which therefore has the potential to contribute to mental health system transformation. The European Commission has defined citizen science as public engagement in scientific research activities, where citizens actively contribute to science either with their intellectual effort, surrounding knowledge, or their tools and resources (14). These activities are often facilitated by information and communication technologies (15, 16). The European Citizen Science Association (ECSA) has published ten principles to serve as best practise guidelines for the application of citizen science within diverse situations and academic disciplines, (17, 18). These are widely used to differentiate citizen science from other research methodologies (19, 20). Citizen science projects have become popular in astronomy, environmental science, biology, ecology, health and medicine (21, 22), where the coproduction of knowledge between researchers, communities and the public can foster improved research outcomes, impact and transparency, and can support data accessibility and utilisation (23). Citizen science enables the public to actively contribute to different aspects of the research process, from conceptualization and data collection to knowledge translation and evaluation (24, 25). Citizen involvement in knowledge mobilisation enhances citizens’ role as constituents and advocates for research outputs, which could potentially motivate decision-makers to engage with researchers in implementing active real-life policies (26).

Mental health research has an established tradition of utilising participatory research approaches, including through survivor and service user-led research, and in the emerging academic disciplines of mad studies (27, 28). In contrast, the use of citizen science as a research approach in mental health is emerging and yet to be established. The application of citizen principles is complex in mental health research, due to possible ethical and legal implications arising from collecting and analysing sensitive information, such as mental health diagnoses and experiences. Citizen science often requires the development of infrastructures to support involvement at scale (15) and it is unclear whether existing citizen science infrastructures are suitable for mental health citizen science. More generally, there is sparse knowledge about citizen science engagement, appropriate research methodologies and standardised guidelines for measuring citizen science metrics and reporting (29, 30).

### Aims and objectives

This systematic review aimed to synthesise published evidence to develop best practice guidelines for conducting citizen science projects in mental health. The objectives were:

#### Objective 1

To investigate the views of researchers and citizen science contributors regarding the use of citizen science projects in mental health in relation to the ten ECSA principles.

#### Objective 2

To identify the ethical and legal issues arising from citizen science approaches in mental health research.

#### Objective 3

To integrate the findings to development of best practice guideline for future citizen science projects in mental health.

## Methods

This review was conducted as part of the Citizen Science To Achieve Co-production at Scale (C-STACS) Study.^1^

### Search strategy and selection criteria

This systematic review was conducted following the Preferred Reporting Items for Systematic Review and Meta-Analysis (PRISMA) guidelines (31). The review protocol was pre-registered (PROSPERO CRD42022316042).

Five sources of data were used. First, a search was conducted across ten electronic databases from inception to the 22nd of March 2022. Due to the cross-disciplinary nature of citizen science, databases from health sciences, social sciences and technology were included. Databases searched were MEDLINE, EMBASE, APA PsycINFO, Cumulative Index of Nursing and Applied Health Literature (CINAHL), Applied Social Science Index and Abstracts (ASSIA), IEEE Xplore, the Social Science Citation Index, Centre for Reviews and Dissemination database, the Cochrane Central Register of Controlled Trials, the Cochrane Library, and Web of Science.

Second, we searched grey literature in citizen science projects blogs and websites (e.g., https://eu-citizen.science/blog, https://blog.scistarter.org/, https://www.spotteron.net/blog-and-news) for discussions about their citizen science activities, and citizen science platforms (e.g., www.patientslikeme.com, www.zooniverse.org) for mental health projects.

Third, we searched conference proceedings from three citizen science-related conferences (citizen science association websites: citizenscience.org/home/events/conferences; European Citizen Science Association: ecsa.citizen-science.net/conference; Engaging citizen science conferences: conferences.au.dk/citsci2022) and two mental health conferences (enmesh.eu/conferences.html; researchintorecovery.com/events/refocus-on-recovery). We then cross-checked for relevant published articles using Google Scholar (first 50 hits).

Fourth, we searched the table of contents from three relevant journals from inception to 30^th^ March 2022: PLOS ONE Citizen Science; British Ecology Journal of Citizen Science; and Journal of Citizen Science: Theory and Practice. Journals were selected to include the main journals specific to citizen science.

Fifth, backward citation tracking was conducted by searching the reference list of all studies included for full-text screening and forward citation tracking of all included documents was conducted using Google Scholar. We did not find any new included papers.

We kept our search strategy broad, combining terms related to citizen science and any type of mental health. The search strategy was co-developed with a subject librarian (AA) and designed to systematically locate all available peer-reviewed research articles, studies published through citizen science platforms and other grey-literature documents providing information on the use of citizen science in mental health. Studies written in any language were considered. The search strategy used is shown in Table 1 and was adapted to each of the relevant databases as required.

**Table 1:** Search strategy.

1	exp citizen science/
2	Citizen science or crowd science or civic science or crowd-sourced science or citizen scientist* or public involvement or community science*.mp. or participatory science. ti,ab.
3	(community adj3 research*).ti,ab.
4	exp Community Participation/
5	exp Patient Participation/
6	1 or 2 or 3 or 4 or 5
7	exp Mental health/
8	exp Anxiety/
9	exp anxiety disorder/
10	exp depression/
11	exp fear/
12	exp Phobic disorders/
13	exp Depressive Disorder/
14	exp Schizophrenia/
15	exp Mental Disorder/
16	exp Mania/
17	exp bipolar disorder/
18	exp Psychotic Disorders/
19	(Mental illness or mental health or mental wellbeing or mental wellbeing or mental disorder*).mp. or mental recovery. ti,ab.
20	(Anxiety or anxious or depress* or fear or phobi* or schizophreni* or mania or manic or bipolar or psychosis).mp. or psychotic.ti,ab.
21	((bipolar or psychiatric or depressive) adj3 disorder*).ti,ab.
22	7 or 8 or 9 or 10 or 11 or 12 or 13 or 14 or 15 or 16 or 17 or 18 or 19 or 20 or 21
23	6 AND 22

We included(inclusion criteria) all studies and documents reporting on (i) citizen science as a research approach to engage public contributors in any form of mental health-related research, (ii) citizen science studies that engaged mental health stakeholders, especially people with lived experience of mental health issues, (iii) the experience of conducting or participating in a citizen science project that is related to mental health, or (iv) the use of citizen science approaches to collect data using any form of study design (quantitative, qualitative or mixed-method, systematic review). We excluded (Exclusion criteria) all studies or documents reporting on the use of other predefined participatory research methods, such as codesign, coproduction or surveys and interviews to involve defined community or study participants in mental health-related research using, studies that were not focusing on mental health. In this review, citizen science was defined as non-scientists participating in the research process to advance science, with ‘citizens’ traditionally defined as inhabitants of a particular locale (without regard to citizenship by legal status) (32). Other relevant definitions considered in differentiating citizen science from other participatory research are included in Table 2.

**Table 2:** Terminology definition.

Citizen science projects	Non-scientists participating in the research process to advance science, with ‘citizens’ traditionally defined as inhabitants of a particular locale (without regard to citizenship by legal status) (32)^.^
Citizen	A member of a community, in the context of this study, this is referring to anyone with lived experience of mental health problems.
Citizen scientist	Anyone who participates in a citizen science project ranges from research question development to the evaluation of the project, this might also include a leadership role. Usually, their participation is as an unpaid volunteer.
Mental health problem	Any emotional or psychological difficulties that make it harder for someone to get on with their lives or that impact a person or those around them. These may be described by a diagnosis made by a professional against a set of diagnostic criteria (e.g., depression, schizophrenia), or they can be self-defined without a formal label.

**Figure 1:**
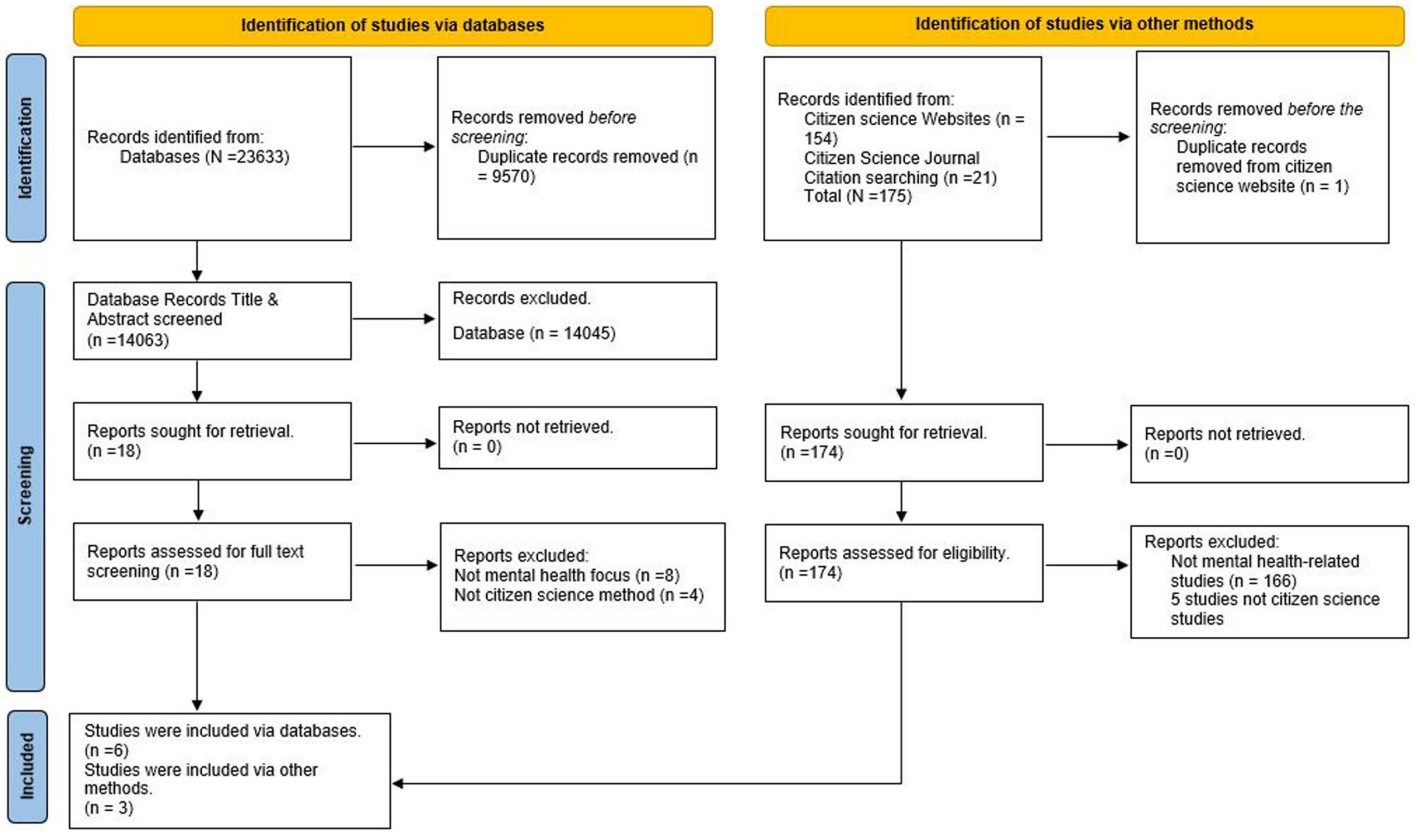
Study selection PRISMA 2020 flow diagram.

The primary reviewer (OT) searched all databases and other sources, identified articles using the search strategy, uploaded to Endnote X9 and de-duplicated them. The systematic review platform Rayyan^2^ was used for eligibility screening. Two independent reviewers (OT & FL) screened titles and abstracts to exclude studies not meeting the inclusion criteria. Discrepancies were resolved through consensus between the two reviewers. The quality of screening was cross-checked by a third reviewer (YK) who independently conducted 10% of the screening. The full-text screening for eligibility was conducted by the first author (OT) for potentially relevant papers. Reasons for exclusion at the full-text screening stage were documented (Figure 1).

### Data extraction

Data from each included publication were independently extracted by two of three reviewers (OT, FL, and YK). Descriptive variables extracted were country of study, clinical population (i.e., type of mental health problem being investigated), study design (e.g., citizen science, randomised control trial), citizen science activity set-up and process details (e.g., time spent by a citizen scientist, name of citizen science platform) and researchers’ report (e.g., limitation and strength of the study, data availability, acknowledgements).

All included studies were treated as primary studies. For example, if an author writes a description of a study that is investigating the use of citizen science on any form of mental health problem, data from the report was extracted regardless of whether the citizen science study was ongoing or completed. The data extraction used a selective approach where the extraction of particular types of data related to the review objectives and questions of interest (33). Data were extracted to a customised and piloted data abstraction form and populated with variables about the study population and phenomena of interest. Double-checking and verification of the data abstraction tool and extracted articles were led by an independent reviewer (MS).

The quality of included studies was not assessed, because the review included a broad range of document types spanning research and non-research articles. Thus, using tools intended to evaluate design quality in research studies was not feasible.

### Data analysis

The publication text was transferred to QSR International Nvivo 12 Pro for data analysis. The evidence synthesis began by organising the data using the ten ECSA principles for citizen science, shown in Table 3, (17). A qualitative evidence synthesis was then conducted through a deductive thematic analysis (34, 35) within each principle whilst preserving the concepts and information that were provided in the included documents. A deductive coding framework was developed (e.g., ‘10. Legal and ethical issues), where relevant adding predefined sub-themes from the ten principles with an ‘Other’ category (e.g.,‘10.1 Copyright’, ‘10.2 Intellectual Property,10.3 Confidentiality, ‘10.4 Attribution’ and ‘10.5 Other’). After familiarising with the data, all documents were independently coded by two reviewers (OT, YK), with frequent consultation with an independent reviewer (MS) to establish a shared understanding of the data in the form of a preliminary coding framework.

**Table 3:** The ten european citizen science association principles.

P1	Citizen science projects actively involve citizens in a scientific endeavour that generates new knowledge or understanding. Citizens may act as contributors, collaborators, or project leaders and have a meaningful role in the project.
P2	Citizen science projects have a genuine science outcome. For example, answering a research question or informing conservation action, management decisions or environmental policy.
P3	Both professional scientists and citizen scientists benefit from taking part.
P4	Citizen scientists may, if they wish, participate in multiple stages of the scientific process.
P5	Citizen scientists receive feedback from the project.
P6	Citizen science is considered a research approach like any other, with limitations and biases that should be considered
P7	Citizen science project data and meta-data are made publicly available and where possible, published in an open-access format.
P8	Citizen scientists are acknowledged in project results and publications.
P9	Citizen science programs are evaluated for their scientific output, data quality, participant experience and wider societal or policy impact.
P10	The leaders of citizen science projects take into consideration legal and ethical issues surrounding copyright, intellectual property, data-sharing agreements, confidentiality, attribution, and the environmental impact of any activities.

The preliminary coding framework consisted of a range of themes describing the operationalisation and definition of each ECSA principle, with sub-themes selected to describe specific reported approaches and experiences of using citizen science. The preliminary framework was iteratively refined and reviewed by all authors. Reviewers maintained reflexivity by consistently discussing to gain a more varied perspective and bringing a broader range of experiences from lived experience, practitioner, and clinical perspectives.

The best practice guidelines were developed by the core review team (OT, YK, MS, SRE). This involved integrating the coding framework by (a) identifying elements specific to mental health research or where citizen science and mental health practises differ, (b) identifying relevant published reporting guidelines and methodologies already used in mental health research, and (c) identifying specific elements to include in reporting studies. The guidelines were reviewed and refined by the wider reviewer team.

### Reviewer team

Consistent with citizen science, the review team consisted of researchers and non-scientist representatives from civic society organisations. The review team brought a range of disciplinary (public health, health research, information specialist, digital research, psychology, geography, sociology) and professional (counselling, clinical psychology, family therapy, nursing, social work) backgrounds, and included multiple civic organisations (specialising in mental health system transformation, survivor-led research, lived experience in research, social media). Some reviewers also identify with having lived experience of mental health problems or mental health distress, ensuring that lived experience is central to the analysis and interpretation.

## Results

### Characteristics of included studies

A total of 23,808 documents (23,633 from databases and 175 from other sources) were identified, and after de-duplication, 14,063 documents titles and abstracts were screened for eligibility. Eighteen publications were found eligible for full-text screening and nine articles were eligible and analysed for this review (36–44); Figure 1.

The studies comprised quantitative (*n* = 3), (36, 39, 43) qualitative (*n* = 1), (37) systematic review (*n* = 1), (38) randomised controlled trial (*n* = 1), (44) project summary report (*n* = 1), (42) mixed-methods (*n* = 1) (40) and citizen science methodology (*n* = 1). (41) All studies were conducted in high-income countries. The terminology used for citizen scientists were participants (*n* = 4), citizens (*n* = 2), citizen scientists (*n* = 1), crowd workers (*n* = 1) and not stated (*n* = 1). Duration of involvement by the citizen scientist in participating in the project ranged from less than 10 min up to 12 months.

Beyond the clinical population, there was no clear pattern in other stakeholders being involved in the citizen science projects, with four studies not identifying any other group, and others identifying involvement from relevant workers/leaders, e.g., school principals (41) or peer coaches. (43) One study reported engagement from multiple groups including families, third-sector organisations, public administration staff, data scientists, city council and social innovators. (42) No platform was used in more than one project. The characteristics of the included studies are described in Table 4.

**Table 4:** Characteristics of included studies (*n* = 9).

ID	Study Author	Country of study	Study aim (summary)	Design	Clinical population	Citizen scientists (*n*)	Platform App/web-based Name
1	Aardoom et al. (36)	Belgium, Netherlands	To investigate whether specific empowering processes and outcomes are experienced on Proud2Bme	Quantitative	Eating disorder	250 Female: 249	Web Proud2Bme
2	Andersen et al. (37)	Denmark	A realist evaluation of how web-based citizen-to-citizen platforms promote belonging and mental health	Qualitative	Mental health	22 Female: 16 Male: 6	Web
3	Naslund et al. (38)	Australia, UK, USA	Assessed potential for using online crowdsourcing methods for behavioural health intervention research with people with serious mental illness	Systematic review	Serious mental illness	1,214 Female: 72%	Not Applicable
4	Washington et al. (39)	USA	Investigated whether a trustworthy crowd of non-experts can efficiently annotate behavioural features needed for machine learning detection of autism spectrum disorder (ASD) in children	Quantitative	Autism Spectrum Disorder (ASD)	Not stated	Web Amazon Mechanical Turk (MTurk)
5	Bliuc et al. (40)	United Kingdom	Examine the role of supportive online social networks in helping people in recovery	Mixed: social network analysis, interviews	Addiction	581 Male: 91%	Web and App Facebook
6	Katapally (41)	Canada	Explored a culturally appropriate digital health initiative in school curricula in rural indigenous communities	Citizen science	Indigenous youth mental health	76	App SmartPlatform
7	Perelló (42)	Spain	Explored the capacity of citizen social science projects to empower participants and provide them with skills to promote collective actions or public policies based on co-created knowledge.	Project Summary Report	Mental health disorder	Project Ongoing	Web SciStarter
8	Simon et al. (43)	USA	Evaluated whether the addition of online coaching from a peer specialist increased participation in an online program featuring educational and interactive modules to promote self-management of bipolar disorder	Quantitative	Bipolar disorder	118 Female: 85	Web MyRecoveryPlan
9	Todd et al. (44)	UK	Tested feasibility and effectiveness of a web-based self-management intervention ‘Living with Bipolar’	Randomised controlled trial	Bipolar disorder	122 Female: 88 Male: 34	Web Living with Bipolar (LWB)

### Thematic analysis in relation to the ECSA principle

The included articles were analysed by synthesising the contents of included studies using the 10 ECSA principles.

#### Principle 1 – involvement

In most cases, the citizen scientist were involved as contributors; providing data, collecting data or completing tasks for the research project (36, 37, 39–42, 44). Data provided by citizen scientists included information such as their demographics, and views on school policies and programs (41). Citizen scientists were involved in research by asking them to collect information about their usage of the citizen science platform, (36, 37) information about their mental health issues (duration of symptoms, treatment status), (36) their personal experiences, (42) and completing and rating tasks. (39) In one study, citizen scientists were also involved as collaborators, acting as co-researchers to co-define, codesign and co-create the citizen social science research (42).

#### Principle 2 – genuine science outcome

The included citizen science projects achieved the goal of a genuine scientific outcome. Six included studies used citizen science approach to investigate primary mental health research (36, 40–44). Three studies evaluated the use of citizen science approaches to answer research questions about mental health belonging, (37) assess citizen scientist trustworthiness in detecting recorded video of children with autism spectrum disorder, (39) and behavioural health intervention research amongst people with severe mental illness (38).

#### Principle 3 (benefits) (objective 1)

##### Both citizen scientists and professional scientists identified benefits

The included studies indicated that citizen scientists identified their participation in their projects to be beneficial and worthwhile. Motivation to participate included forming social relationships, connectivity and joining a group or community (37, 40). The benefits of participating in citizen science projects included: citizen scientists being empowered, feeling better informed about their condition, promoting their mental health, well-being and acceptance of their illness, and increasing their help-seeking behaviour through sharing and exchanging information, and improved self-esteem (36, 37). Other benefits arising from participation included: personal enjoyment, relaxation from reading about other people’s personal storeys and mental health experiences; finding recognition, help and exchanging information with fellow peers about their mental health issues; enhancing their coping skills; decreasing stress and stigma, improved sense of hope; improving their mental health condition, quality of life, wellbeing, recovery and social function; and (for youth) allowing them to focus on their strengths and build resilience against their mental health challenges such as substance abuse, depression and anxiety (36, 38, 40, 41). The least reported benefit of involvement was citizen scientists discussing their mental health with a health professional (36). The only harm identified was the considerable amount of commitment and investment of time and energy required to participate which could be burdensome on the citizen scientists (41).

The professional scientists and researchers identified some benefits of citizen science participation based on its impact on science and citizen scientist. In terms of scientific benefits, conducting and designing citizen science projects was a way of demonstrating a commitment to a shared and collective research approach that is inclusive of researchers, communities and individuals (42). Integrating citizen science, community participatory research action approach with smartphone usage in research all contributed to overcoming traditional research constraints, such as increased participant recruitment and retention, data collection and analysis, interventions, and knowledge translation (41). For example, online crowdsourcing indicated a feasible and acceptable approach for reaching people with severe mental illness, to confirm their diagnosis, self-report and collate their health outcomes, and deliver adaptable interventions (38). Citizen scientists were found to be efficient and accurate in completing tasks (39). In terms of benefits for citizen scientists, participation in projects was seen by researchers as a promising tool for social connectivity, (37) and empowerment of individuals with mental health disorders, encourages participants to take control of their lives and manage their health conditions (36). Health outcomes for citizen scientists were reported as positive (38). For example, participating in an online community helped to build bridging recovery capital, meaning participants had access to a recovery-supportive online community, engaged with the outside world inclusive of community stakeholders, and helped participants to create a sense of hope in their recovery journey (40). An e-community offers qualitatively good information and provides interactive involvement and space for individuals to enjoy themselves (36).

Moreover, these benefits are achievable with the presence of supportive initiatives such as IT support, and professional facilitators to facilitate activities on the citizen science platforms (37). The main challenge identified by researchers was the time required to engage with the public, communities, and institutional management to promote citizen science projects (41). This engagement process was time-consuming but important; researchers reported that the benefits of citizen science projects are dependent on the interest of citizen scientists, the level of good information provided by citizen scientists and interactive involvement between researchers and citizen scientists (36).

#### Principle 4 (stages of participation)

Most citizen scientists in the included studies participated in data collection, (36, 41, 43, 44) and analysis of data that were allocated by professional researchers (39, 41, 42). For example, citizen scientists (referred to as crowd workers) were assessed for their trustworthiness and task completion in analysing the behavioural features needed for accurate machine learning detection of common Autism Spectrum Disorders in children (39). Similarly, the CoACT study proposed to allow citizen scientists to participate in the analysis and interpretation of data by establishing of citizen’s parliament that will include all mental health stakeholders who cocreated the study (42). Some projects involved citizen scientists before data collection, for instance, youth citizen scientists, people with mental health problems and their families, academics, third sector organisations and public administrators cocreated the study design (41, 45). In another, people with bipolar disorder contributed to the design of the crowdsourcing platform and the citizen science activities before the study implementation (43). Citizen scientists also participated in the dissemination and communication of citizen science results (41, 42).

#### Principle 5 (feedback)

Most projects did not report any aspect of feedback, either citizen scientists receiving project feedback or researchers providing feedback on the use of citizen science data. Only one study reported that evidence generated in their project was disseminated and translated into a societal outcome and knowledge mobilisation, which amplified the voice of indigenous youths in the community (41). The study did not provide further information on how this dissemination and translation of outcome was conducted.

#### Principle 6 (research approach)

All included studies reported on the limitation and biases of using citizen science as a research approach. A potential bias reported was in the way researchers frame citizen science task instructions for citizen scientists, thus influencing the citizen scientist’s responses towards the direction of the researcher’s desired outcomes and outputs (39). Another study discussed that the level of citizen scientist engagement and participation on project platforms is dependent on the number of participating citizen scientists, and concluded that citizen scientist engagement did not predict research outcomes (43). The impact and capacity of the citizen science project to support mental health is strongly dependent on citizen’s abilities and opportunities to develop quality relationships during the citizen science project activities, that satisfy their mental health belongingness needs and transform their engagement into positive relational outcome (37). Another study reported that citizen scientists feeling empowered through participating in a citizen science project could be influenced by other unknown external factors (36). The involvement of different stakeholders could potentially have an impact on the project’s quality, efficiency and expected outcome, due to conflicting interests and influence such as varied implementing academic institution strategic plan, political institution, social dynamics and citizen scientist expectation (41). One challenge reported in a systematic review of citizen science studies was the issue of hostile messages posted by some citizen scientists on the project platform discussion boards, posing potential threats to self and distress for other citizen scientists, this was reported in two out of the seven studies included the systematic review (38). Another study reported a similar experience of citizen scientists posting threatening and negative comments, unsolicited health tips and advice on the citizen science platform, but reported that a designated project facilitator was tasked with moderating and deleting such comments on the citizen science platform (36).

#### Principle 7 (data availability)

None of the nine included studies reported public availability of their data. Moreover, one study stated that the study data were not made publicly available because citizen scientists did not consent to data sharing due to privacy and ethical concerns (37). Another study stated that study data could be requested from the authors (39). Concerning open access to data, seven studies published their project in open access publication journal (37–43) and two were not open access (36, 44).

#### Principle 8 (acknowledgement)

Five studies acknowledged their citizen scientists by thanking them for their participation in the acknowledgements section as an un-specified group (i.e., not by name). (37, 39–41, 44). One project also acknowledged other stakeholder groups such as non-governmental organisations. (41) The remaining four studies included in this review did not report nor acknowledge the participation of their citizen scientists (36, 38, 42, 43). It is noteworthy, that two studies did not report any form of citizen scientist acknowledgement because one was reporting on a systematic review of citizen science projects (38) and the other on a pre-implementation project report (42).

#### Principle 9 (evaluation)

All included studies reported the strengths, limitations, and outcomes of their citizen science projects in relation to their scientific output, data quality, participant experience and wider societal or policy impact.

##### Scientific output

All studies or documents included in this review generated a scientific output in the form of publishing a research publication or project summary report. Three of the included studies reported and published the process and result outcome of the citizen science project (41, 43, 44). One of the studies was a realist evaluation of twenty-seven citizen scientists about their experiences in using the citizen science platform to improve mental health wellbeing (37). A study conducted an in-depth interview with citizen scientists and a social network analysis of the citizen science platform to capture the dynamics of interactions between citizen scientists (40). Aardom et al. (2014) examined and reported the extent to which citizen scientists are empowered through their participation in a citizen science project, and measures the correlation between citizen scientists’ empowerment and their health outcome (36). The trustworthiness of citizen scientists and their efficiency in completing a citizen science task was assessed and published (39). The CoAct project published a monograph, detailing the citizen science project proposal and expected outcome (42).

##### Data quality

Two studies reported that achieving a high-quality and reliable citizen science data requires the participation of a large number of diverse citizen scientists, (39, 40). In some instances generating quality citizen science data requires project-specific training for citizen scientists (39). Increased participation was associated with higher levels of motivation and commitment from citizen scientists (43). Data quality was improved by separating citizen scientist chat rooms according to different project groups to reduce data contamination, (43) or by moderating contributions (36). The prevention of data contamination was important, to separate participants that were involved in the Recovery plan only from others that were communicating with peer support coaches (43).

##### Participant experience

Citizen scientists were able to use and participate in citizen science projects through online platforms, though high dropout rates were identified and reported by researchers. Researchers reported that citizen scientists dropped out of the project due to the following reasons: lack of time and commitments from citizen scientists due to busy schedules; (41, 44) technical difficulties with platforms and internet access; (41) significant life events; (44) inability to engage online and preference for face-to-face interactions; (38) and lack of acknowledgement (40). Citizen scientists identified two barriers to participating in citizen science projects, limited participation time due to other commitments and personal lives busy schedules and technical difficulties in using online platforms (38). Effective engagement of participants was dependent on participants’ feeling of being endorsed and supported, having a sense of community belonging, and acknowledgement of their contributions (36, 40).

##### Wider societal or policy impact

A study reported that the involvement of individuals and other stakeholders in citizen science has the potential to be a catalyst for collective action, leading to an impact on healthcare policy, mental healthcare self-management, and digital health with specifics on the use of digital tools for citizens with mental health issues and their families (42). Projects providing platforms in which individuals can share their mental health experiences and find recognition for their experiential knowledge, contribute to the development of best practises in e-health (36). The participatory approach of citizen science projects creates opportunities for scalable and replicable digital health interventions (41).

#### Principle 10 (ethical and legal issues) (objective 2)

Two primary researchers included in this review reported to have obtained ethical approval before the commencement of their research (41, 44). Three studies reported obtaining informed consent from their citizen scientists through a protected application before any engagement and data collection (41, 43, 44). Three broad ethical issues were identified: security, personal information, and safeguarding issues. Security issues related to ensuring communication with citizen scientists is secure, and that access to platforms is password-protected. Personal information was addressed by not collecting any personal or clinical information, and not recording participants’ IP addresses (36). Where eligibility to participate in the citizen science project was restricted to individuals having the mental health issue of interest, de-anonymisation of data was of safeguarding concern, for example ensuring that collected data could not be linked with any social media sites and participant’s social media profile (40). One project addressed this concern by making the content of the project publicly available to anyone visiting without registration but requiring citizen scientists to register on the platform before any access to the project’s forum and chat activities (36). Safeguarding issues were addressed by the researcher monitoring the projects forum and discussion boards, where citizen scientist engages with each other (38, 43). For instance a study reported that to protect participants privacy all communications between peer coaches and citizen scientists occurred within the secure website where the study is being conducted (43). Building strong partnerships with the citizen scientists community based on equity, respect, co-ownership of data and co-produced knowledge translation was identified as an important element of the successful implementation of mental health citizen science research (43). All included studies did not report on citizen science participation attribution and the environmental impact of the citizen science project activities.

#### Development of best practice guideline (objective 3)

The small number of studies that met the inclusion criteria for this review indicated the novelty in the use of citizen science as a research approach in mental health research. ECSA health working group has notably stated that the use of citizen science in health research is relatively under-represented, despite its promising potential (46). All studies included in the review did not discuss following any reporting guidelines in the conduct of their research. Evidence has shown that reporting and assessing health research practises and experiences assists in developing best practice guidelines and ensures quality (47). It also assists in improving reporting, accuracy, completeness and transparency of the most important aspects of health research studies (48). To initiate a discussion on best practice principles of mental health citizen science research built on our review’s results, ECSA principle and common principles that guide participatory mental health research, we inferred and propose the following best practice guideline for conducting and reporting a mental health citizen science research– Table 5. The best practice guideline highlights the key concepts that should be in place for the conduct and reporting of a mental health citizen science project.

**Table 5:** Best practice guidelines for citizen science and mental health.

	ECSA Principle	Mental health-specific considerations	Reporting Relevant reporting guidelines, methodologies or specific elements to report
1	Involvement	Citizen scientists include people with lived experience of mental health issues (service users, non-service users, patient councils, PPI representatives, informal carers, clinicians, and the public). Involvement of people with lived experience of mental health and stakeholders can occur from the initial development of the study to feedback stages. Roles of people with lived experience in mental health in citizen science project could include but not limited to contributor, collaborator, and project leader. Citizen science studies should adopt approaches to minimising barriers to involvement.	Guidelines: GRIPP2 (49) Specifics: state the level (e.g., contributor, collaborator, project leader) and type (e.g., crowdsourcing, participatory, extreme citizen science) of involvement
2	Genuine science outcome	Citizen science is particularly applicable to addressing knowledge gaps that can: transform mental health research to policy and practice; be addressed using designs which can be co-developed and coproduced with citizen scientists; involve person-centred and community-focussed designs; lead to outcomes of relevance to citizens. Involve key stakeholders (e.g., policy makers, relevant stakeholders) in the codesigning and coproducing to ensure that research leads to change.	Research question framework: PICO, (50) SPICE, (51) SPIDER (52) Specifics: state the knowledge gap including ethical considerations
3	Benefits	Identify planned benefits for contributors and researchers from the outset. Contributor benefits may include empowerment, engaging with research, attribution, social connectivity and building a community. Participation payment to citizen scientists is not the norm, though more active roles (e.g., project leader) should be reimbursed. Evaluate contributor and researchers benefits as part of the project.	Specifics: evaluation of contributor’s views on benefits, and demonstration that benefits were not solely for the researchers.
4	Stages of participation	The most common participation stages are data collection and analysis. Involvement in earlier (e.g., co-design) and later (e.g., dissemination) stages of citizen science mental health project is also possible. For instance, Peer researchers can support involvement at all stages, as can leadership board membership. Participation at all stages of the project should be encouraged by researchers.	Methodologies: generative codesign framework, (53) EBCD (54) Specifics: state the citizen participation at each research stage, even if none.
5	Feedback	Plan and build in a co-developed feedback approach from the start, to inform citizen scientists about (a) key findings and (b) use of findings.	Methodology: data visualisation (55, 56) Specifics: report the feedback process, and report the feedback approach built into data generation and validation.
6	Research approach	Consideration should be given to safeguarding issues, to protect people with lived experience of mental health. This should include but not limited to including potential harms arising from (a) interactions between contributors or between contributors and the research team, (b) health-related advice being offered by contributors and (c) mismatched project expectations between contributors and researchers.	Specifics: The title or abstract should use the term ‘citizen science’. Critical reflections on the strengths and limitations of the method should be reported.
7	Public data availability	Open data (making publicly available anonymised datasets in an appropriate data repository) is consistent with citizen science principles but rarely implemented, partly due to privacy concerns. Early co-creation of a data management plan with contributors is important. Ensure data protection legislation (e.g., GDPR (57, 58) and relevant local (e.g., funder, research organisation, clinical service) information governance policies are followed. The open-access publication is the norm.	Specifics: Name the secure citizen science platform (e.g., SciStarter, Zooniverse, PatientsLikeMe) and data repository (e.g., UK Data Service, OpenAIRE, data.gov).
8	Acknowledgement	Any presentation (e.g., website, talk, academic paper) of findings should acknowledge: (a) all contributing citizen scientists as an un-named group; (b) with their permission, individual named contributors who have made significant contributions at any research stage; and (c) with their permission, named representative groups. Wording for this acknowledgement should be agreed between contributors and researchers early in the project. Approaches should be considered for individuals to indicate they wish to be named as contributors and to involve contributors in co-authoring publications.	Specifics: IJMCE author guidelines (59) should be met by contributor co-authors. Acknowledgements section should always thank contributors as a group, and individuals by name where relevant and with permission.
9	Evaluation	Evaluation should cover scientific outputs, data quality (e.g., diversity of contributors, participation challenges such as digital exclusion, and moderation challenges), participant experience (time commitment, technology useability), researcher experience (how challenges were resolved, adaptability and flexibility in the planning and implementation of the project) and wider society or policy impact.	Specifics: In addition to traditional mental health research evaluation, also impact of the project on contributors and researchers, and knowledge mobilisation approaches.
10	Legal and ethical	If no personal (e.g., sociodemographic, or clinical information) information is collected, the standard citizen project approach of not obtaining project-specific informed consent may be appropriate. Where personal information is collected, either in-person or online consent should be obtained from citizen scientists. Technology security, and information governance approaches including anonymisation, and safeguarding should be addressed. Co-development of a data management plan is recommended, describing how data will be collected, analysed, reported and shared, and who will own the data and any resulting intellectual property. A collection of IP addresses or moderation of contributions should be explicit. An accessible summary should be available for contributors to view. There should be support provided for the platform moderation by the researchers such as debriefing, and training.	Specifics: statements about ethical approvals, data ownership and data sharing (e.g., Creative Commons licence).

## Discussion

To the best of our knowledge, this is the first systematic review evaluating the use of citizen science in mental health research. We synthesised the reported views of citizen scientists and researchers regarding mental health citizen science projects, including possible ethical and legal issues. The citizen science approach was applied in different mental health research for data collection, analysis, and delivery of interventions with a focus on citizen scientist empowerment, mental health promotion, recovery, and self-management. Our findings indicated that the use of the citizen science approach in mental health research is still in its infancy stages but feasible, because of the small number of eligible studies for this review (only nine studies). Our findings showed that most citizen scientists were involved as contributors and participated in the projects mostly during the data collection stages. Our results showed that citizen science projects encompass the collaboration and partnership between professional scientists and citizen scientists, including taking part in scientific activities, ranging from data contribution to comprehensive participation and project co-creation (60, 61). This supports evidence that citizen science as a research approach has the potential to actively engage and involve the public at every stage of research, beyond data collection (62). Though evidence shows that the level of involvement and participation of citizen scientists is dependent on the purpose and objective of the project design, type of research question, and available resources (63). The characteristics of citizen science projects also influence the intensity of citizens’ engagement, participation and geographical sampling frame (64).

Citizen science has the potential to foster citizen engagement and democratise knowledge generation, especially when integrated with established participatory research approaches in health research (25, 65). Citizen science has different frames for the conduct of research, as a result of its inclusivity, flexibility and adaptiveness at stages of the research process (15); Haklay 2018; (66) stated that participation is the key term that differentiates citizen science from other forms of participatory research, such as participatory action research, public involvement that is commonly used in mental health research. This “participation” could occur in spectrums based on citizen scientists’ engagement in contribution to democratic science, he noted that “participation,” however, remains open to multiple interpretations and arguably abuse by researchers (66). Moreover, studies have encouraged the use of the citizen science approach alongside other participatory approaches such as community-based participatory research, co-design and coproduction as a means fitting the approach within a wide variety of disciplines and practises (15, 25). A common example in the social science field is the involvement of civil society organisations and researchers to codesign solutions to societal problems, through a shared, open and reflexive research process (15). Since citizen scientists can participate in leadership roles in projects, in our opinion, citizen science research has the potential to challenge epistemic exclusion, by encouraging research participation from people who are otherwise excluded from research processes due to difficulties engaging in social settings through technology. Moreover, researchers must take caution in the use of this approach, not to worsen exclusion, for example by introducing barriers to participation from people who struggle with the use of technology. Propagation of research ownership by researchers who are already empowered against citizen scientists must be avoided. As the use of the citizen science approach evolves, frameworks that support the integration of the citizen science approach and other participatory methods, and address prospective challenges, such as ensuring active engagement, citizen science activity digital tools, and data integration is urgently required (15, 25).

Our review indicated that citizen scientists included in the studies enjoyed participating in citizen science projects because of their associated benefits. Evidence has shown that participating in citizen science assists in improving scientific literacy, and knowledge reasoning skills and changes public attitudes to science (67, 68). We noted that designing and promoting active citizen science engagement and participation requires a time commitment from researchers, citizen science project designers and citizen scientists. Past research emphasised that ensuring multiple stakeholder perspectives, inputs and citizen scientists’ engagement in citizen science projects, requires planning which is time-consuming both for researchers and the stakeholders (69). Most of the studies included in our review obtained ethical approval and informed consent for the implementation of their research projects. Moreover, the key ethical issues identified in this review were data protection and security, personal informal and safeguarding issues such as the identification of offensive posts on citizen science platforms though they were removed by project facilitators. The studies reporting these issues stated that mitigating such harm requires early planning and moderation of citizen science platforms. Similarly, Rayland et al. (2023) highlighted the increased use of online modes of delivering mental health-related interventions and the use of moderators as a key success to creating a safe and positive community (70). However, further research is required on the use of online platforms for mental health peer support and the role and mediation experiences of platform moderators (70). Similarly, the emotional safety of researchers and participants in participatory research is an ongoing discussion, that requires flexibility on the part of researchers and participants about how and what data is shared (71). A community-based participatory research study identified that mutual respect, management of the power relationships between researchers and participants, and moderation of forums were approaches to safely involving community members in all phases of their research process (72).

The ECSA principle does not guide the management of possible harm that participation in citizen projects poses on citizen scientists, especially people with mental health problems. Additionally, our study found that there was no consensus on the way mental health citizen science research should be reported and conducted, despite available guidelines such as the ECSA principles. All studies included in the review reported their project results and evaluation diversely, without any inference to following any guiding implementation or reporting principle. Despite the growing consensus of citizen science potential, the strategies for the implementation and standardised guidelines for measuring citizen science metrics and reporting are sparse (29, 30). Therefore, in starting a discussion around the ethical, legal and safeguarding issues that might arise as a result of online citizen science projects we are proposing the best practice guideline for mental health citizen science projects (Table 5). The use of a best practice guideline is important in building trust with communities and support for citizen science project designers (63).

All the studies included in our review were conducted in high-income countries. This supports evidence that there is sparse usage of citizen science approach in low-income countries, which is attributed to researchers and the public unfamiliar with the approach, and cultural differences about the use of online and technology platforms for research (73, 74). In our opinion, given that some aspects of citizen science are often implemented through technology mechanisms, digital exclusion may be a barrier to the adoption of citizen science in settings with limited access to technology and digital forms of research. A study discussed the framework for decolonising digital citizen science by enabling equitable participation in citizen science projects, especially amongst digitally excluded populations (75). Therefore, we are suggesting that further research be conducted on how best to promote inclusivity and exclusion of citizen science projects especially for digitally excluded populations. Future research should consider understanding measures of decolonising citizen science perspectives, and the use of citizen science in low– and middle-income settings.

We reported in our study that most projects did not report any aspect of feedback, either by the citizen scientists receiving project feedback or researchers providing feedback on how the citizen science data were used. The ECSA principle encouraged the provision of feedback to citizen scientists about how their data is used and processed. The provision of research feedback to citizen scientists promotes possible future involvement between citizens and professional scientists, ensures the use of relevant outcome measures, (76) and improves overall research quality. Lack of trust has been associated with poor mental health issues, and the use of robust feedback mechanisms between institutions (including researchers) and the public can strengthen or weaken public trust in institutional science (77, 78). For instance, daily COVID-19 pandemic debriefings were associated with building populations’ institutional trust and policy acceptance (79, 80).

In our opinion. There is the potential for ‘citizen science’ to be a contentious term in mental health, due to its implied acceptance of the terms and assumptions of science by people with lived experience of mental health, and the potential for inadvertent exclusion of people who do not identify as citizens. One study has highlighted that the meaning of ‘citizen’ is different between many Western countries and the rest of the world and that the terms ‘science’ and ‘research’ are more frequently associated with the novelty of findings in the West compared to the East Asia such as Japan where it does not communicate novelty (81). Hence, citizen science as a term may not be universally transferable, researchers are beginning to promote inclusivity by rebranding it as “community science” to avoid the term “citizen.” (82). Another possible contention is the discussion around financial remuneration for citizen science activity participation. Most citizen science project participation is voluntary, though this is not occasionally the case (83). This is different from the current practice in mental health research which encourages remuneration of research participants for their time when participating in any public engagement or involvement activity, in the form of cash or gift voucher. Despite the challenges that the use of citizen science is assumed to pose for health research, it is expected that citizen science may yield better knowledge, empower communities and improve community health (64).

The strengths of this review include the use of multiple sources both large databases, citizen science project-specific platforms and websites to search for eligible studies, the involvement of analysts bringing diverse perspectives, and the originality of this first-in-field review. The review’s inclusion and exclusion criteria allowed for different studies including grey literature to be included in the review, if eligible. However, a limitation of our study was that most of the included studies were found through empirical research databases, and most of the grey literature websites and platforms screened lacked sufficient information required for inclusion in the review. Another limitation of our study is that the reasons for non-inclusion at title and abstract screening stage were not recorded. To maximise comprehensiveness, we included studies investigating Autistic Spectrum Disorder, which in some settings would not be considered a mental health condition. A stricter interpretation will be warranted as the evidence base diversifies. Also, only two studies reported on the societal outcome and impact of using the citizen science approach in mental health. Our proposed best practice guidelines were inferred based on nine documents, indicating the development of knowledge is in its early stages. We anticipate that the proposed best practice guideline will evolve as experience increases in using citizen science approaches in mental health research. Development in other applications of citizen science indicates this is likely to happen. For example, best practice guidelines have been developed to guide scientists and practitioners in the development and use of digital citizen science applications (84).

## Conclusion

Our review found nine studies that used a citizen science approach to mental health. To set this in context, a recent scoping review found only 81 articles that have used the citizen science approach across all health research, they were predominantly in physical activity and nutrition (85). The use of citizen science in mental health research is developing but still needs to address specific issues, e.g., consent, safeguarding and stigma. Compared with its use in natural sciences, its use in mental health research requires some adaptation, careful consideration of the consent process, impact and benefits monitoring, feedback and data ownership is needed. The best practice guidelines provide a preliminary defensible theoretical foundation for further research in mental health citizen science studies. This will assist mental health researchers, citizen science researchers and citizen scientists to co-develop more mental health-tailored projects. Citizen science has the potential to bring a more nuanced understanding of the views of professionals and citizens on the conduct of, and participation in, mental health research. This will support the development of mental health citizen science research as an important new methodology and emerging research approach to harness experiential expertise. In conducting health-related citizen science research, researchers, communities and policymakers must ensure to collaborate, co-create and share ownership in all aspects of research, towards translating data into comprehensible and actionable output at the population level (86).

## Data availability statement

Data collected for the study, and a data dictionary defining each field in the set, will be made available to others; this data are what were extracted for use in this review from the included study, this includes (data identifiers, data dictionary, ECSA principle definition); and the study protocol. The requested data will be made available with publication as an Appendix.

## Author contributions

OT and MS conceptualised the study, and OT wrote the first draft of the report. OT, FL, and YK extracted and analysed data. MS had access to and verified all data. All authors contributed to the article and approved the submitted version.

## Funding

This work is funded by UK Research and Innovation (UKRI) Citizen Science Collaboration Grant (Funder reference: BB/V011707/1). MS and SR-E are supported by the NIHR Nottingham Biomedical Research Centre. MS and SR-E are supported by the NIHR Nottingham Biomedical Research Centre (NIHR203310).

## Conflict of interest

The authors declare that the research was conducted in the absence of any commercial or financial relationships that could be construed as a potential conflict of interest.

## Publisher’s note

All claims expressed in this article are solely those of the authors and do not necessarily represent those of their affiliated organizations, or those of the publisher, the editors and the reviewers. Any product that may be evaluated in this article, or claim that may be made by its manufacturer, is not guaranteed or endorsed by the publisher.

## Footnotes

^1^ researchintorecovery.com/c-stacs

^2^ https://www.rayyan.ai
