# Supplementary material for: Best practice guidelines for citizen science in mental health research: systematic review and evidence synthesis
Source: Front Psychiatry. 2023 Sep 8;14:1175311. doi: 10.3389/fpsyt.2023.1175311 (PMC10515389; doi:10.3389/fpsyt.2023.1175311)
Supplement: Supplementary file 1 [file Table_1.DOCX]

**Supplementary material**

Appendix 1: Full search terms for OVID database for MEDLINE, PsychINFO.

| 1. | exp citizen science/ |
| --- | --- |
| 2. | (Citizen science or crowd science or civic science or crowd-sourced science or citizen scientist* or Public involvement or community science*).mp. or participatory science.ti,ab. [mp=title, abstract, original title, name of substance word, subject heading word, floating sub-heading word, keyword heading word, organism supplementary concept word, protocol supplementary concept word, rare disease supplementary concept word, unique identifier, synonyms] |
| 3. | (community adj3 research*).ti,ab. |
| 4. | exp Community Participation/ |
| 5. | exp Patient Participation/ |
| 6. | exp Mental health/ |
| 7. | exp Anxiety/ |
| 8. | exp anxiety disorder/ |
| 9. | exp depression/ |
| 10. | exp fear/ |
| 11. | exp Phobic disorders/ |
| 12. | exp Depressive Disorder/ |
| 13. | exp Schizophrenia/ |
| 14. | exp Mental Disorder/ |
| 15. | exp Mania/ |
| 16. | exp bipolar disorder/ |
| 17. | exp Psychotic Disorders/ |
| 18. | (mental illness or mental health or mental wellbeing or mental well being or mental disorder*).mp. or mental recovery.ti,ab. [mp=title, abstract, original title, name of substance word, subject heading word, floating sub-heading word, keyword heading word, organism supplementary concept word, protocol supplementary concept word, rare disease supplementary concept word, unique identifier, synonyms] |
| 19. | (anxiety or anxious or depress* or fear or phobi* or schizophreni* or mania or manic or bipolar or psychosis).mp. or psychotic.ti,ab. [mp=title, abstract, original title, name of substance word, subject heading word, floating sub-heading word, keyword heading word, organism supplementary concept word, protocol supplementary concept word, rare disease supplementary concept word, unique identifier, synonyms] |
| 20. | ((bipolar or psychiatric or depressive) adj3 disorder*).ti,ab. |
| 21. | 1 or 2 or 3 or 4 or 5 |
| 22. | 6 or 7 or 8 or 9 or 10 or 11 or 12 or 13 or 14 or 15 or 16 or 17 or 18 or 19 or 20 |
| 23. | 21 and 22 |

**Appendix 2: List of studies excluded at full-text screening stage for published Articles, with brief reasons.**

|  | **Name of Study and Author** | **Brief Reason for Exclusion** |
| --- | --- | --- |
| 1 | Al-Laith A, Alenezi M. Monitoring people’s emotions and symptoms from Arabic tweets during the COVID-19 pandemic. Information. 2021 Feb 19;12(2):86. | Study was not citizen science method |
| 2 | Andersen TO, Dissing AS, Varga TV, Rod NH. The SmartSleep Experiment: Evaluation of changes in night-time smartphone behavior following a mass media citizen science campaign. Plos one. 2021 Jul 21;16(7):e0253783. | Study was not focusing on mental health |
| 3 | Cao H, Cooper DG, Keutmann MK, Gur RC, Nenkova A, Verma R. Crema-d: Crowd-sourced emotional multimodal actors dataset. IEEE transactions on affective computing. 2014 Jul 8;5(4):377-90. | Study was not focusing on mental health |
| 4 | Dorwart RA, Meyers WR, Norman EC. Effective citizen participation in mental health: comparative case studies. Public Health Reports. 1979 Jun;94(3):268. | Study was not citizen science method |
| 5 | Frei A, Dalla Lana K, Radtke T, Stone E, Knöpfli N, Puhan MA. A novel approach to increase physical activity in older adults in the community using citizen science: a mixed-methods study. International Journal of Public Health. 2019 Jun;64(5):669-78. | Study was not focusing on mental health |
| 6 | Hensel JM, Shaw J, Ivers NM, Desveaux L, Vigod SN, Bouck Z, Onabajo N, Agarwal P, Mukerji G, Yang R, Nguyen M. Extending access to a web-based mental health intervention: who wants more, what happens to use over time, and is it helpful? Results of a concealed, randomized controlled extension study. BMC psychiatry. 2019 Dec;19(1):1-0. | Study was not citizen science method |
| 7 | Hinckson E, Schneider M, Winter SJ, Stone E, Puhan M, Stathi A, Porter MM, Gardiner PA, Dos Santos DL, Wolff A, King AC. Citizen science applied to building healthier community environments: advancing the field through shared construct and measurement development. International Journal of Behavioral Nutrition and Physical Activity. 2017 Dec;14(1):1-3. | Study was not focusing on mental health |
| 8 | Patel DI, Winkler P, Botello J, Villarreal J, Puga F. The citizen scientist: Community-academic partnerships through Translational Advisory Boards. Patient education and counseling. 2016 Dec 1;99(12):2087-90. | Study was not focusing on mental health |
| 9 | Pykett J, Chrisinger B, Kyriakou K, Osborne T, Resch B, Stathi A, Toth E, Whittaker AC. Developing a Citizen Social Science approach to understand urban stress and promote wellbeing in urban communities. Palgrave Communications. 2020 May 6;6(1):1-1. | Study was not focusing on mental health |
| 10 | Williams CR, Burnell SM, Rogers M, Flies EJ, Baldock KL. Nature-based citizen science as a mechanism to improve human health in urban areas. International Journal of Environmental Research and Public Health. 2021 Dec 22;19(1):68. | Study was not focusing on mental health |
| 11 | White JJ, Mathews A, Henry MP, Moran MB, Page KR, Latkin CA, Tucker JD, Yang C. A crowdsourcing open contest to design pre-exposure prophylaxis promotion messages: protocol for an exploratory mixed methods study. JMIR research protocols. 2020 Jan 3;9(1):e15590. | Study was not focusing on mental health |
| 12 | Prior K, Salemink E, Wiers RW, Teachman BA, Piggott M, Newton NC, Teesson M, Baillie AJ, Campbell S, Stapinski LA. Acceptability and Co‐Development of an Online Cognitive Bias Modification Intervention for Emerging Adults With Hazardous Alcohol Use and Social Anxiety: A Mixed Methods Study. Alcoholism: Clinical and Experimental Research. 2020 Nov;44(11):2283-97. | Study was not citizen science method |

**Appendix 3: List of citizen science platforms and other grey literature websites.**

| https://eu-citizen.science/blog |
| --- |
| https://eu-citizen.science/projects |
| https://blog.scistarter.org/ |
| https://scistarter.org/ |
| https://www.spotteron.net/blog-and-news |
| https://www.spotteron.net/apps/global-community-science-projects |
| https://www.spotteron.net/apps/regional-community-science-projects |
| https://www.spotteron.net/apps/horizon-2020-horizon-europe |
| https://www.spotteron.net/apps/finished-community-science-projects |
| https://www.patientslikeme.com |
| https://www.zooniverse.org/ |
| https://conferences.au.dk/citsci2022 |
| https://ecsa.citizen-science.net |
| https://citizenscience.org/home/events/conferences |
| http://enmesh.eu/conferences.html |
| *PLOS ONE Citizen Science* |
| British Ecology Journal of Citizen Science |
| Journal of Citizen Science: theory and practice |

**Appendix 3: Codebook for data extraction from included documents.**

|  | **Category** | **Definition** |
| --- | --- | --- |
| **A** | **Characteristics of the included document** | |
| A1 | Reference | Included articles reference. |
| A2 | Study title | The title of the included study. |
| A3 | Authors contact email | The contact details of the corresponding author. |
| A4 | Publication Type | The type of document is either a full report, systematic review, Abstract or news brief or scientific brief report. |
| A5 | Study Aim | Verbatim text of the aim of the included study. |
| A6 | Study design | The type of means that data were collected in the included study i.e. qualitative, or quantitative study. |
| A7 | Country | The country where the study was conducted. |
| A8 | Data collection period | The period of data collection as reported by the researcher i.e. data collection starts and end period. |
| A9 | Mental health problem | This is the mental health problem of interest in the included study. |
| A10 | Time spent by citizen scientist | Verbatim report of the total or range of time that was reported that the citizen scientist participated in the citizen science activities. |
| A11 | Name of funder | The name of the funder of the project as reported verbatim by the researchers. |
| A12 | Type of funder | Whether this was a research funder, government organisation or any other kind of funder. |
| A13 | Declaration of conflict of interest | Declaration of conflict of interest by the authors, as stated verbatim in the included articles. |
| A14 | Sociodemographic | The demographics of the citizen scientist as presented by the researchers such as age, sex, gender. |
| A15 | Type of technology | The type of technology or platform that was used in conducting the citizen science project. |
| A16 | Consent process | Verbatim declaration of whether a consent form was taken or not from the citizen scientist. |
| **ECSA principle data analysis coding** | | |
| **P1.0** | Involvement | Verbatim reporting on how the citizen scientist was involved in the citizen science project. |
| *P1.1* | *Collaborators* | Whether citizen science scientists were involved as collaborators for the study such as from the study inception. |
| *P1.2* | *Contributors* | Whether citizen scientists were involved mainly in the project activities. |
| *P1.3* | *Project Leaders* | Whether the citizen scientist was stated to be part of the project leader or not. |
| **P2.0** | Genuine science outcome | Reference to any study aims and objectives that is focused on science. |
| **P3.0** | Benefits | Any reported benefits of conducting or participating in a citizen science project and activities as reported by researchers or quotation by a citizen scientist. |
| *P3.1* | Citizen scientist views | Reported benefits or harms of participating in a citizen science project by a citizen scientist. |
| *P3.2* | Professional scientist views | The reported benefit of citizen science or harm of conducting citizen science project, as reported by the researchers. |
| **P4.0** | Stages of Participation | The reported stages of participation for the citizen scientist in the citizen science project. |
| *P4.1* | *Gathering and analysis of data* | Report or assumption that the citizen scientist was only involved in data collection or data collection and analysis. |
| *P4.2* | *Method design* | Report that the citizen scientist was involved in the design of the citizen science project. |
| *P4.3* | *Results communication* | The report in the document that citizen scientist was involved in the analysis, and dissemination of the project output. |
| **P5.0** | Feedback | Any form of a report indicating how citizen scientists were feedback on their participation and contribution to the project activities. |
| **P6.0** | Research Approach | The indication that citizen science was treated as a research study with its limitation and biases were adjusted for and controlled where possible. This is extracted from the strength and limitation section of the articles, if available. |
| *P6.1* | *Biases* | Report on the biases that might have influenced the citizen science project, citizen scientist activities and how this was considered and controlled for. |
| *P6.2* | *Limitation* | Report of the limitation of the study because of using the citizen science approach, reported by the researchers. |
| **P7.0** | Public Availability of Data | Report on the availability of data that was contributed by the citizen scientist or that was analysed by the citizen scientist. Details on how the data can be accessed, where it is saved and how it is being managed. Details of who has access to the data and how the data is anonymised. |
| **P8.0** | Citizen Scientist Acknowledgement | Details in the document of citizen scientists being acknowledged for their contributions and how they were acknowledged and attributed for their participation and involvement in the study. How the citizen scientist requested to be acknowledged. |
| **P9.0** | Evaluation | Report on how the impact and quality of data generated through the citizen science activities and citizen scientist participation experiences were measured, monitored and evaluated. |
| *P9.1* | *Data Quality* | The report in the document discusses the quality of the data, validation of data and measures that were put in place to ensure data quality and control checks. |
| *P9.2* | *Participants Experience* | Reports or quotations in the documents on the participant’s experiences of participating in the citizen science activities and collaboration, either positive or negative. |
| *P9.3* | *Scientific Output* | Any report on the scientific outcome that the citizen science activities have produced in the form of the journal article, publications within the communities, etc. |
| *P9.4* | *Wider society and/or policy impact* | Any report on the wider society and policy impact that resulted because of the citizen science activities and the results that were generated. |
| **P10.0** | Legal and Ethical issues | Report on the legal and ethical issues and how it was managed during the conduct and implementation of the citizen science project and activities. What are the ethical and legal issues that were envisaged and how the risk was managed. |
| *P10.1* | *Attribution* | Report on how the citizen scientist wanted to be attributed for their contributions, and what arrangement was made to ensure that citizen scientist were attributed for their contributions. |
| *P10.2* | *Confidentiality* | Report on what confidentiality arrangement was made during the citizen science contribution, during the data management and analysis, this also covered the informed consent process and how informed consent was received and asked. |
| *P10.3* | *Copyright and intellectual property* | Report on the arrangement between researchers and citizen scientist on data ownership, copyright and intellectual property, what repository is used and how citizen scientist has control over their data and what level of control. |
| *P10.4* | *Data Sharing* | Report on how the data provided by the citizen scientist is shared, whether the contributors permit for the data to be shared, and the process or protocol of sharing data, what data sharing guideline guides the sharing of the citizen scientist contributions. |
